# Telecoupled impacts of livestock trade on non-communicable diseases

**DOI:** 10.1186/s12992-019-0481-y

**Published:** 2019-07-01

**Authors:** Min Gon Chung, Jianguo Liu

**Affiliations:** 1 Center for Systems Integration and Sustainability, Department of Fisheries and Wildlife, Michigan State University, East Lansing, MI 48823 USA; 2 Environmental Science and Policy Program, Michigan State University, East Lansing, MI 48824 USA

**Keywords:** Livestock trade, Livestock consumption, Meat consumption, Non-communicable diseases, Telecoupling

## Abstract

**Background:**

Non-communicable diseases (NCDs)—chronic human health problems such as cardiovascular diseases linked to poor diets—are significant challenges for sustainable development and human health. The international livestock trade increases accessibility to cheap animal products that may expand diet-related NCDs worldwide. However, it is not well understood how the complex interconnections among livestock production, trade, and consumption affect NCD risks around the world.

**Method:**

Our global dataset included 33 livestock products (meat, offal, and animal fats) in 156 countries from 1992 to 2011. We employed path analysis to uncover how livestock trade contributes to diet-related NCDs and identify underlying environmental and socioeconomic factors of livestock trade. Then we performed trend analyses to investigate long-term changes in livestock production and trade at a country level.

**Results:**

We found that livestock consumption through livestock import increased diet-related NCD risks. This was especially true in developing countries, which in general were not well prepared in terms of policies for NCD risk reduction, and where there was a lack of funding to implement the policies. Population size and income level were the main factors affecting global livestock import activities.

**Conclusions:**

Our results suggest that new governance structures to incorporate separate international efforts, improved national policies, and bolstering individual efforts are needed to decrease NCD risks, particularly in developing countries.

**Electronic supplementary material:**

The online version of this article (10.1186/s12992-019-0481-y) contains supplementary material, which is available to authorized users.

## Background

The increasing risks of non-communicable diseases (NCDs) have been recognized as significant health challenges around the globe [1–4]. In 2011, the United Nations (UN) General Assembly adopted a political declaration to reduce and prevent NCDs [5]. In 2013, the World Health Organization (WHO) Global Action Plan on NCDs dealt with both behavioral and metabolic risk factors and considered the social and economic impacts of NCDs [6]. Additionally, the UN Sustainable Development Goals (SDGs) rank NCDs as one of the core components that link human health and sustainable development [7–9].

Countries with continuous population and income growth have experienced a rapid nutrition transition toward consuming more livestock products [1, 4, 10, 11]. But many countries cannot meet consumer demand for livestock through domestic production, and thus they are increasingly dependent on imported energy-dense animal products [12–14]. Livestock imports may lead to increases in the consumption of meat and animal fats, and thus NCD risks, especially cardiovascular diseases, type II diabetes, cancer, and chronic respiratory diseases [1, 15–18]. Consumption of livestock products such as fatty and processed meat is one of the major dietary risk factors for NCD incidence and mortality [4, 16, 19, 20]. For example, high meat consumption stands out as a strong contributor to colorectal cancer [21]. Additionally, diets high in processed meat are moderately linked with an increased risk for cardiovascular disease [19, 20, 22] and diabetes [23]. Although some countries have raised concern about livestock trade (e.g., cheap fatty meat) in relation to increases in diet-related NCDs [24–26], little research has quantified or thoroughly explained how the complex interrelationships among livestock production, trade, and consumption affect diet-related NCDs worldwide. Additionally, global livestock supply chains from producers to consumers make it complicated to quantify the impacts of livestock trade on NCDs across countries [27].

To fill this knowledge gap, our research objective was to investigate how livestock trade affects livestock consumption and the risks of NCDs, as well as which socioeconomic and environmental factors contribute to livestock production and trade. We also examined the role of agents (e.g., farmers and consumers) in facilitating or preventing livestock trade and consumption across countries. To guide our research, we used the integrated framework of telecoupling (socioeconomic and environmental interactions over distances) [28], which helps explain complex interconnections among livestock production, trade, consumption, and the risks of NCDs simultaneously. This telecoupling framework allows analysis of the socioeconomic and environmental interconnections among two or more coupled human and natural systems over long distances [29]. This framework has been applied to a variety of important issues, such as trade (of food, energy, sand, and forest products) [30–33], land use and land cover change [34–36], species migration [37], tourism [32, 38], water transfer [39, 40], urbanization [41], wildlife transfer [32], foreign direct investment [42], payment for ecosystem services [43, 44], knowledge transfer [32], conservation [43–45], economic development [46], and fisheries [47, 48]. This is the first time, however, that this framework has been used in the context of livestock production, trade, consumption, and human health.

Specifically, we evaluated the major components of the framework—effects (impacts of the trade on NCD risks and mortality) and causes (reasons behind the trade)—across 156 countries where livestock production and consumption occur. We included 33 livestock products that were divided into three groups (meat, offal, and animal fats). For detailed analysis, we chose four focal countries (Brazil, China, the UK, and the USA) to represent different groups for livestock production, trade, consumption, and risks of NCDs around the world. We evaluated flows (amounts of traded livestock) and agents (entities that facilitate trade) in these four focal countries.

## Methods

### Components of the telecoupling framework

The telecoupling framework highlights flows, causes, effects, and agents in the coupled systems [28]. Countries in this study are the *coupled human and natural systems* where humans interact with natural components (e.g., pastures and meadows) [49]. *Flows* are the movement of livestock products among different countries. *Effects* indicate the human health impacts of livestock consumption. *Causes* refer to various factors that influence livestock flows. And *agents* are entities that facilitate or prevent flows (movement of livestock products in this study) directly and indirectly [28], such as livestock producers, consumers, and traders. By including these components, we examined the effects of livestock trade on livestock consumption and the risks of NCDs, investigated factors that contribute to livestock production and trade, and identified the role of agents that facilitate livestock trade and consumption simultaneously.

### Coupled systems

This study identified the interconnections among livestock production, trade, and consumption, and their impacts on diet-related NCDs in 156 countries (see Additional file 1). Each country can be viewed as a coupled human and natural system. By selecting these 156 countries, we examined the complex interrelationships among livestock production, trade, consumption, and NCD risks at the global level.

Then we obtained a more detailed understanding of the complex interconnections of these factors by selecting four focal countries (Brazil, China, the UK, and the USA) that represent different livestock production, trade, and consumption levels. These four focal countries accounted for nearly 55% of global livestock production and 30% of global livestock trade. Whereas Brazil and the USA were net food exporters, China and the UK were net food importers [50]. Additionally, while per capita GDP-PPP (Purchasing Power Party) in Brazil, the UK, and the USA increased approximately 35–45% from 1990 to 2011, it increased 546% in China. Urban populations in Brazil and China have increased four and five times respectively from 1961 to 2011, but urban populations have increased only slightly in the UK and USA. As developing countries, Brazil and China experienced rapid urbanization and income growth from 1961 to 2011. The UK and USA represent developed countries with stabilized urbanization and income growth rates.

### Data collection

We obtained relevant data from the FAOSTAT [50], the World Bank [51], and the Global Health Data Exchange (GHDx) [52]. The dataset covers 156 countries from 1961 to 2011, but some data are only available beginning in the 1990s. We divided the 156 countries into developed (*n* = 46) and developing (*n* = 110) countries according to the World Bank’s classification (see Additional file 1) [51].

We included 33 livestock products and 58 crops (see Additional file 2). Livestock products were largely divided into meat, offal, and animal fats. Using nutritive factors [53], food production and trade data were converted from the mass unit (tonne) to kilocalories (kcal). Because kcal per mass unit differ among and within animal products (e.g., between pork meat and offal), the mass unit cannot represent the role of livestock production and trade in livestock consumption and its impact on NCD risks. We selected 33 livestock products from FAOSTAT for which nutritive factors were available. Using FAO Food Balance Sheets, we also chose 58 crop items used for livestock feed. FAOSTAT provided data on food production, trade quantities, and other agricultural factors such as pasture and meadow areas [50]. Socioeconomic data such as population and per capita GDP-PPP came from the World Bank [51].

The GHDx provided disability-adjusted life years (DALYs), the number of deaths, and mortalities per 100,000 people associated with NCDs at five-year intervals, 1990–2015 [52]. The Global Burden of Disease project of the GHDx estimated diet-related NCD DALYs, the number of deaths, and age-standardized mortality using a comparative risk assessment framework [18]. We concentrated on risk factors regarding diets high in red and processed meat, because our research focused on livestock consumption and its impact on NCD risks. The livestock diet-related NCD deaths included deaths from colon and rectal cancer, diabetes mellitus, and ischemic heart disease [18]. We also included DALYs to represent the prevalence of livestock diet-related NCDs in each country. In a given population, the DALY is a measure of overall disease burden, expressed as the number of years lost due to disability or premature death from a certain disease (colon and rectal cancer, diabetes mellitus, and ischemic heart disease in this study) [54].

### Flows of livestock trade

To understand which countries play an important role in global livestock production and trade, we mapped livestock trade flows into the four focal countries using FAO detailed trade matrices from 1992 to 2011. We averaged livestock export products for each exporter and drew livestock import flows from the exporters to the four countries.

### Path analysis

We selected 156 countries in the period from 1992 to 2011 for path analysis, because data availability starting in the early 1990s was better than for the previous decades. Along with the collapse of the Soviet Union, many new countries became independent around this time and environmental and socioeconomic data were available after 1992 from international organizations. Selecting the period from 1992 to 2011 allowed us to minimize the number of missing values for statistical analyses and therefore establish reliable statistical models.

We performed path analysis using Mplus Version 7.4 [55]. Path analysis allows the identification of relations among observable variables [56, 57]. Path analysis also allows the quantification of the *causes* and *effects* of the telecoupling components simultaneously. Endogenous variables were domestic livestock production, livestock export and import kcal, per capita livestock consumption, diet-related NCD DALYs, the number of deaths, and age-standardized mortality. Exogenous variables included environmental (pasture and meadow areas) and socioeconomic factors (per capita protein supply of animal origin, per capita GDP-PPP, and population size). Data were available for 156 countries, and we calculated the mean annual factors from 1992 to 2011 for each country. At the country level, the GHDx provided DALYs, the number of deaths, and age-standardized mortality rates per 100,000 people due to diets high in meat in 1995, 2000, 2005, and 2010 as the sum of the three NCDs (colon and rectal cancer, diabetes mellitus, and ischemic heart disease). We averaged diet-related NCD DALYs, the number of deaths, and age-standardized mortality from 1995 to 2010.

We constructed structural models to examine the interconnections between livestock production, trade, consumption, and NCD risks. First, we hypothesized that socioeconomic and environmental factors affect the amount of livestock production and trade. These in turn may affect per capita livestock consumption. Finally, per capita livestock consumption may influence diet-related NCD DALYs, the number of deaths, and age-standardized mortality, while controlling for per capita GDP-PPP, population size, and pasture and meadow areas. For the sake of clarity, we also performed alternative path analysis with meat production, trade, and consumption, instead of total livestock products.

To achieve linearity and normality, we performed a log transformation on all variables. We used the Maximum Likelihood (ML) method to estimate coefficients. We also reported the *χ*^2^, the standardized root mean square residual (SRMR), and the comparative fit index (CFI). For each model, the SRMR should be close to or lower than 0.08, and the CFI should be close to or higher than 0.90 [58].

### Trend analysis

To support the results of path analyses and investigate long-term trends, trend analyses were done in the four focal countries from 1961 to 2011. We summed up the total crop and livestock supply (kcal) and per capita livestock consumption (kcal/capita/day) for each of the four countries. We calculated both crop and livestock supply, because crops are required to feed livestock. To calculate crop and livestock supply, the domestic production was subtracted from total import calories and added to export calories. Using FAO Balance Sheets, we classified the total crop supply into six categories of crop utilization: food supply, feed, seed, waste, processing, and other uses. We also calculated livestock consumption in terms of three subcategories in the four countries: meat, offal, and animal fats. Specifically, meat consumption was divided into bovine, pork, and poultry to determine consumption patterns in the four countries.

## Results

### Flows of livestock trade in the four focal countries

**Fig. 1:**
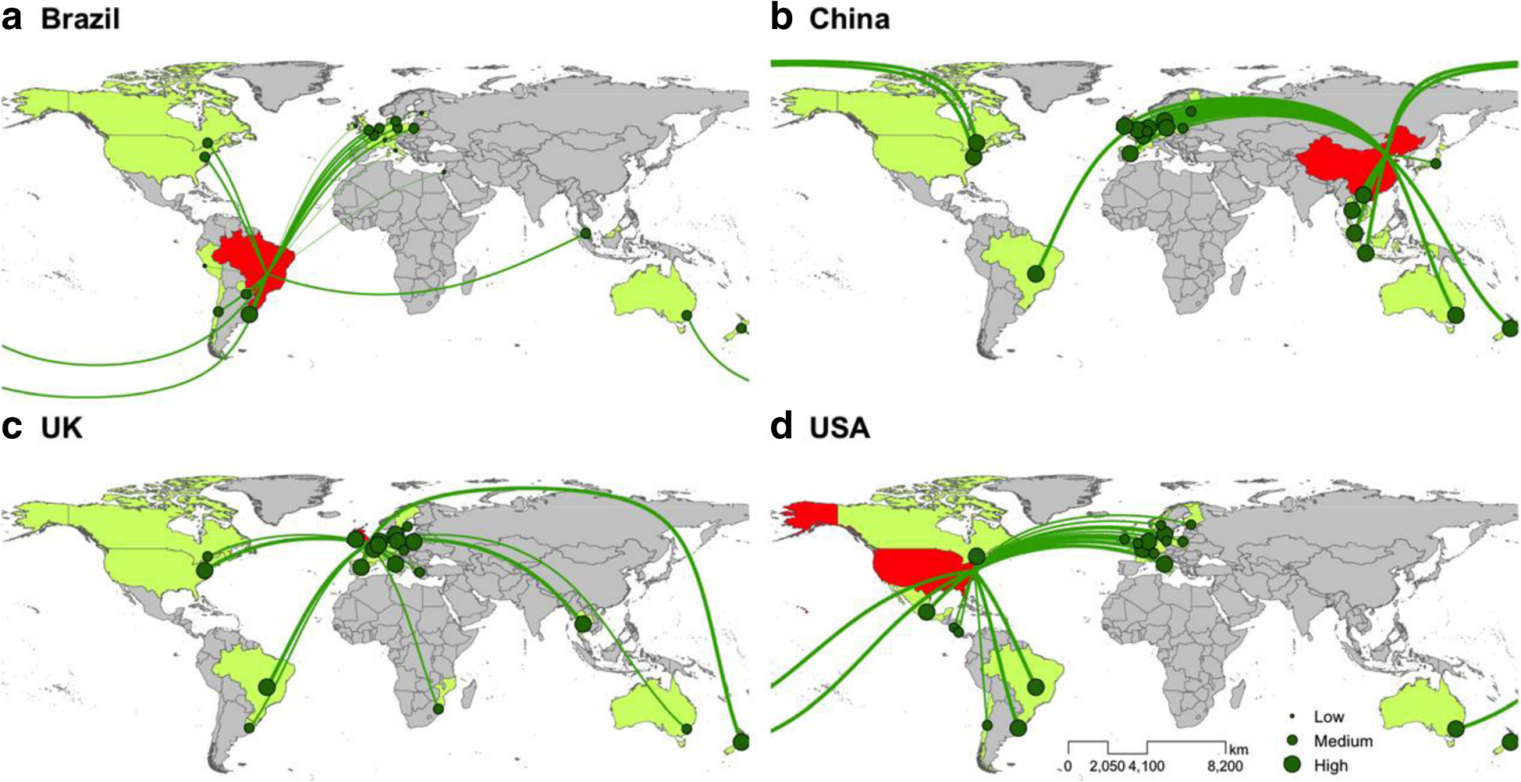
Flows of livestock imports in the four focal countries from 1992 to 2011. **a** Brazil, **b** China, **c** the UK, and **d** the USA: Red color indicates livestock importing countries (receiving systems), and green indicates livestock exporting countries (sending systems). Gray indicates spillover systems that have not exported livestock products to receiving systems but are affected by the trade of livestock products between sending and receiving systems. The size of the green circles shows the relative calories of livestock exported. The map was generated by the Telecoupling-GeoApp [70]. Data source: The FAOSTAT [50]

Livestock trade forms the flows of livestock products between countries. China and the UK particularly depended more on imported livestock than Brazil and the USA. China imported livestock commodities mostly from distant countries—the USA, New Zealand, and Brazil—while the UK (Fig. 1) mainly imported livestock from Western European countries such as Ireland, Denmark, and the Netherlands.

### Effects of livestock consumption on NCD risks via livestock trade

The flows of livestock trade affected livestock consumption and NCD risks directly and indirectly—changes in per capita livestock consumption resulting from differences in livestock production and trade affected diet-related NCD risks across regions. First, while countries with high livestock production had high livestock consumption, the countries that imported large amounts of livestock also tended to have high rates of consumption (Table 1). In the alternative model, the amounts of both meat production and import were also positively associated with meat consumption (see Additional file 3). Rapidly developing countries experienced positive relationships between livestock import and livestock consumption. Those countries could not meet the new demand for meat even though livestock production was high, and they turned to other countries to make up the difference. For example, although China produced the largest amounts of livestock in the world (148 trillion kcal per year or 36.9% of global livestock production from 1992 to 2011), it had a hard time meeting rapidly increasing livestock consumption demands with its domestic production. Thus, China imported the largest amounts of livestock in the world (4.7 trillion kcal per year or 8.6% of global livestock imported).

**Table 1:** Path analysis of the relationships between livestock consumption and diet-related NCD risks through livestock production and trade from 1992 to 2011

Path analysis	Unstandardized coefficient (S.E.)
Dependent variable: Livestock production (kcal)	
Protein supply of animal origin (g/capita/day)	1.482** (0.184)
Pasture and meadows (km^2^)	−0.068* (0.031)
GDP-PPP per capita (2011 $ const.)	−0.006 (0.103)
Population (persons)	1.190* (0.048)
Dependent variable: Livestock export (kcal)	
Protein supply of animal origin (g/capita/day)	4.119** (1.131)
Pasture and meadows (km^2^)	−0.060 (0.190)
GDP-PPP per capita (2011 $ const.)	1.208 (0.633)
Population (persons)	1.429** (0.292)
Dependent variable: Livestock import (kcal)	
Protein supply of animal origin (g/capita/day)	0.897* (0.292)
Pasture and meadows (km^2^)	−0.051 (0.049)
GDP-PPP per capita (2011 $ const.)	0.761** (0.163)
Population (persons)	0.633** (0.075)
Dependent variable: Livestock consumption (kcal/capita/day)	
Livestock Production (kcal)	0.370** (0.035)
Livestock Export (kcal)	0.005 (0.006)
Livestock Import (kcal)	0.049* (0.024)
Pasture and meadows (km^2^)	0.053** (0.015)
GDP-PPP per capita (2011 $ const.)	0.222** (0.043)
Population (persons)	−0.539** (0.042)
Dependent variable: Disability-adjusted life years from high (years)	
Livestock consumption (kcal/capita/day)	1.557** (0.164)
Pasture and meadows (km^2^)	0.023 (0.043)
GDP-PPP per capita (2011 $ const.)	0.518** (0.115)
Population (persons)	1.085** (0.065)
Dependent variable: Number of deaths from diets high in meat (persons)	
Livestock consumption (kcal/capita/day)	1.616** (0.171)
Pasture and meadows (km^2^)	−0.011 (0.044)
GDP-PPP per capita (2011 $ const.)	0.404* (0.120)
Population (persons)	0.978** (0.068)
Dependent variable: Age-standardized death rate from diets high in meat (per 100,000 people)	
Livestock consumption (kcal/capita/day)	0.589** (0.096)
Pasture and meadows (km^2^)	0.016 (0.025)
GDP-PPP per capita (2011 $ const.)	0.184* (0.067)
Population (persons)	0.080* (0.038)
*χ* ^2^	149.851
df	14
CFI	0.941
SRMR	0.026

Values in parentheses are standard errors

All variables are log transformation variables, except the index of NCD risk factors

* *P* < 0.05, ** *P* < 0.001

Second, per capita livestock consumption was positively associated with diet-related NCD DALYs, the number of deaths, and mortality at a country level (Table 1). In the alternative model, per capita meat consumption was also positively associated with diet-related NCD DALYs, the number of deaths, and mortality (see Additional file 3). Population size and per capita GDP-PPP played essential roles in diet-related NCD deaths and mortality.

**Fig. 2:**
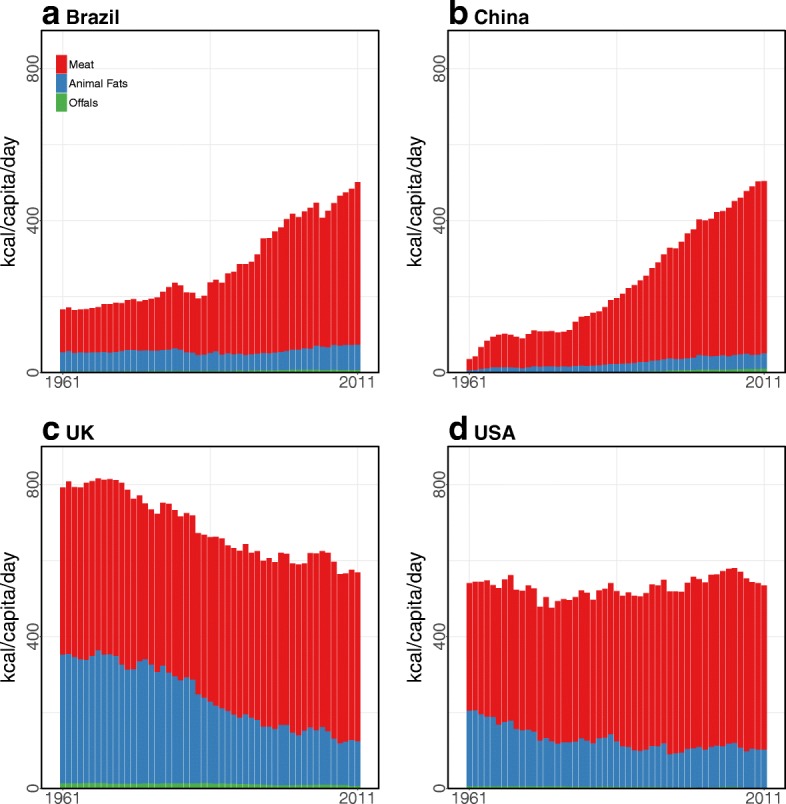
Livestock consumption by subcategories from 1961 to 2011: **a Brazil** increased meat consumption after the mid-1980s. **b** Meat consumption in **China** led to increases in total livestock consumption. **c** Animal fat consumption in the **UK** slightly decreased, but meat consumption stabilized. **d** The proportion of livestock consumption in the **USA** was stable. Data source: The FAOSTAT [50]

In the four focal countries, although the UK and USA consumed more livestock than Brazil and China and thus suffered higher NCD risks, the UK and USA had lower rates of change in NCD DALYs, deaths, and mortality than Brazil and China (Fig. 2 and Table 2). On the one hand, from 1995 to 2010, Brazil and China respectively increased livestock consumption 37.1 and 53.4% while increasing 183.9 and 302.6% in terms of diet-related NCD DALYs (Table 2). DALY rates in Brazil and China also increased 133.1 and 268.1% from 1995 to 2010. In the same period, Brazil’s and China’s NCD deaths also increased 195.3 and 310.7% due to diets high in meat. Diet-related NCD mortality in Brazil and China increased 142.4 and 275.5%, respectively. On the other hand, the UK decreased livestock consumption by 4%, while the USA increased 4.2% from 1995 to 2010. However, both the UK and USA decreased both diet-related NCD DALYs (35 and 0.9%, respectively) and DALY rates (40 and 14.7%) from 1995 to 2010 (Table 2). The UK and USA also decreased the number of diet-related NCD deaths by 38.4 and 16.1%, and diet-related NCD mortality decreased 43.2 and 27.8%, respectively. Although NCD DALY rates in developing countries like Brazil and China were lower than those in developed countries, rates of diet-related NCD deaths and mortality increased more rapidly than in developed countries.

**Table 2:** Per capita livestock consumption, disability-adjusted life years (DALYs), number of NCD deaths, and NCD mortality due to diets high in meat in our four focal countries

	Brazil	China	UK	USA
Livestock consumption (kcal/capita/day)				
1995	353	328	599	518
2010	484	503	568	534
Change rate (%)	37.1	53.4	**−**4.0	4.2
1995–2010	419	413	600	550
DALYs (years)				
1995	45,325	92,811	293,625	1,665,482
2010	128,673	373,648	190,965	1,650,528
Change rate (%)	183.9	302.6	**−**35.0	**−**0.9
1995–2010	88,365	235,207	234,547	1,773,067
DALY rate (years per 100,000 people)				
1995	27.71	7.60	506.48	625.13
2010	64.59	27.97	303.86	533.07
Change rate (%)	133.1	268.1	**−**40.0	**−**14.7
1995–2010	47.39	18.03	391.61	611.93
Number of deaths (persons)				
1995	1212	1997	14,804	74,133
2010	3579	8200	9113	62,177
Change rate (%)	195.3	310.7	**−**38.4	**−**16.1
1995–2010	2402	5060	11,593	72,974
Age-standardized death rate (deaths per 100,000 people)				
1995	0.74	0.16	14.50	20.08
2010	1.29	0.39	19.37	25.26
Change rate (%)	142.4	275.5	**−**43.2	**−**27.8
1995–2010	1.29	0.39	19.37	25.26

**Fig. 3:**
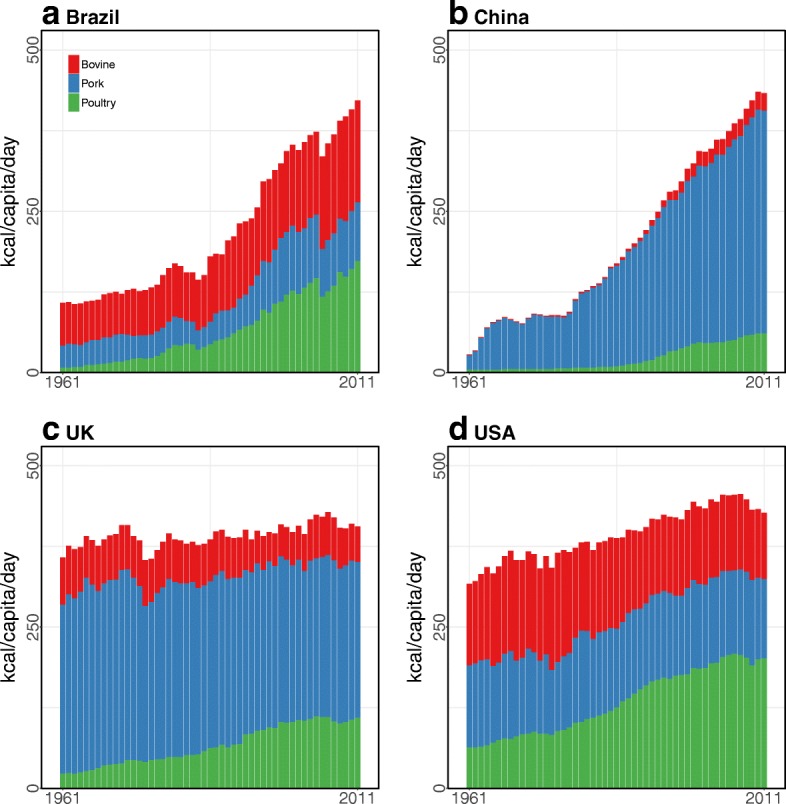
Meat consumption of bovine, pork, and poultry from 1961 to 2011: **a Brazil** exponentially increased its poultry consumption. **b** Increased meat consumption in **China** came from pork. **c** The **UK** stabilized its total meat consumption but consumed more poultry. **d** Although the **USA** consumed less bovine and pork meats, poultry consumption in the USA increased over the past five decades. Data source: The FAOSTAT [50]

In developed countries, the UK (568 kcal in 2011) and USA (534 kcal in 2011) had similar per capita livestock consumption rates, but they had different livestock consumption patterns. For example, the USA was highly dependent on poultry meats over the past five decades, while over half of the meat consumption in the UK was from pigs (Fig. 3). In addition, from 1992 to 2011, the decreases in animal fats (−36%) and bovine meat (−11%) in the UK led to a decreased livestock consumption (Figs. 2 and 3).

### Factors related to livestock production and trade

Socioeconomic and environmental factors were related to the changes in livestock production and trade that affected livestock consumption and NCD risks. In the 156 countries examined in this study, the main determinant of livestock production and trade was population size over the study period (Table 1). While population size was positively associated with livestock production and trade, income level was not significantly associated with livestock production. This is because livestock production rapidly increased in highly populated countries. Developing countries with high populations such as Brazil, China, and India increasingly led this trend.

Livestock export activities were positively associated with per capita protein supply of animal origin, because high domestic livestock supplies were a prerequisite for the availability of livestock products for export [15]. Livestock import activities were also positively associated with per capita protein supply of animal origin, because many developed countries such as Germany and the UK import livestock products that they cannot produce yet demand. In addition, countries with higher population and income levels imported more livestock from abroad, as income levels and population size led to people eating more meat and animal fats [1, 4]. Along with the increase of livestock imports in developed countries, developing countries have also increasingly imported large amounts of livestock while experiencing rapid economic and population growth. Results from the alternative model for meat production, trade, and consumption had the same significant factors, except for the relationship between per capita GDP and meat export (see Additional file 3). Countries with higher income levels tended to export more meat products abroad.

### Agents for livestock production and trade

Agents facilitate or prevent livestock production and trade across regions, and therefore can influence livestock consumption as well as NCD risks. Although the USA had the second-largest amount of livestock production (12.5% of global livestock production from 1992 to 2011), the number of farmers in the USA was only 0.5% of farmers in China and 23% of those in Brazil (Table 3). We used the number of all farmers to represent producers, as this research included both crop feed and livestock supplies. Although the UK and USA had fewer farmers, consumers in these two countries consumed a higher number of calories of livestock products. China had many more farmers and consumers than the other three countries. Furthermore, consumers in Brazil and China spent higher proportions of their overall expenditures on livestock products than those in the UK and USA.

**Table 3:** Agents, household food expenditures, and government expenditures on health in our four focal countries (data sources: The FAOSTAT [50], WHO [68], The World Bank [51], and IMAP [69])

	Brazil	China	UK	USA
Farmers, 1000 persons (2011)	10,495	504,523	463	2410
Consumers, 1000 persons (2011)	196,935	1,344,130	63,259	311,583
Livestock consumption, kcal/capita/day (2011)	501	504	568	534
Protein supply of animal origin, g/capital/day (2011)	50	37	58	69
Food and beverage companies, numbers (2010)^a^	31	153	48	303
Household food expenditure, % (2008)	24.1%	33.0%	8.5%	5.9%
Household livestock products expenditure, % (2008)	12.7%	13.4%	3.7%	2.4%
Government expenditure on health, % (2012)	7.6%	12.5%	16.1%	19.9%

^a^ According to IMAP [69], food and beverage companies were classified into the farming, processing, and distribution sectors; companies’ sectors were identified according to Bloomberg’s classification: (https://www.bloomberg.com/research//common/symbollookup/symbollookup.asp)

Although China had the lowest NCD mortality due to diets high in meat (0.39 deaths per 100,000 people from 1995 to 2010) among the four countries, the number of consumers in China was the highest among the four focal countries. Diet-related NCD DALYs and the number of deaths in China increased 302.6 and 310.7% from 1995 to 2010, respectively (Table 2). Since China has not effectively established and enacted NCD policies for its 1.3 billion consumers, the number of people in China exposed to potentially severe diet-related NCD risks with the increased livestock consumption was much higher than in other countries. Additionally, Brazil and China had lower government expenditures for healthcare than the UK and USA, and thus the low government expenditures contributed to high NCD mortality in Brazil and China (Table 3).

## Discussion

Based on the telecoupling framework, this study quantified the interrelationships among livestock production, trade, and consumption as well as their impacts on diet-related NCDs simultaneously. Many countries have both produced more livestock products domestically over time and also imported more livestock products from other countries. Our results show that livestock imports had a positive association with rising NCD risks via the increases in livestock consumption, particularly in highly populated developing countries that had rapid income growth. Population size and income level were the main factors affecting the increases in livestock consumption and diet-related NCD risks. Results from the alternative model for meat products also confirmed the positive relationship between meat imports and rising NCD risks resulting from the increases in meat consumption.

Although developing countries had lower NCD risks (e.g., lower DALY rates) than developed countries, they had rapid growth in rates of diet-related NCD deaths and mortality. The different change rates may be due to different healthcare infrastructures and health expenditures in developed and developing countries, as NCD deaths and mortality vary depending on healthcare access. Governments in developing countries struggled with both malnutrition reduction and NCD risk control with limited budgets and thus were challenged to achieve both goals simultaneously. Most health promotion initiatives are practiced in developed countries [2]. In developing countries, less-funded health initiatives appeared to be ill equipped to cope with their population’s new livestock diet-driven NCD risk. The different change rates in NCD risks and mortality can also be caused by different social determinants (e.g., education and employment levels), human behaviors related to livestock consumption, and cultural and historical backgrounds [11, 59].

**Fig. 4:**
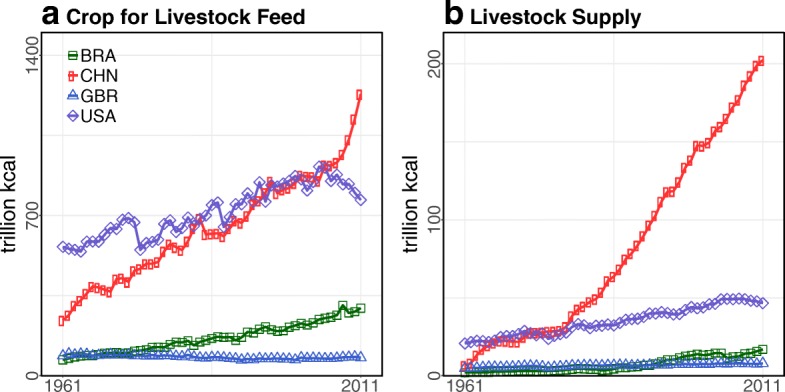
Net crop and livestock supply from domestic production and trade in Brazil, China, the UK, and the USA: **a** **Crops for livestock feed**. The USA and China used more crop calories for animal feed than Brazil and the UK. China rapidly increased livestock feed after 2007. **b** **Net livestock supply**. Brazil and China have exponentially increased livestock supply since 1961. Brazil exceeded livestock supply in the UK after 1993, and China exceeded livestock supply in the USA after 1972. Data Source: The FAOSTAT [50]

We also examined the role of agents (producers and consumers) in livestock production, trade, consumption, and diet-related NCDs. Since the globalization of the livestock trade generates complex heterogeneous networks [60], other agents such as policymakers, trade and agriculture ministers, and transnational corporations may influence the amount of livestock trade and consumption across and within countries in important ways [4, 11, 25, 61]. For example, although China had greater crop feed and livestock supplies than the USA after 2001 (Fig. 4), the number of food companies in China was lower than in the USA (Table 3). This may indicate that China’s infrastructure for processing and distributing food is poor compared to that of the USA. Consequently, China may distribute food inefficiently and waste more during distribution. Whereas the USA lost less than 1% of crops through waste in the food supply chain (e.g., storage and transportation), China lost about 3–4% of its total crop supply through waste after the 2000s (see Additional file 4).

Because livestock production, trade, and consumption are deeply intertwined worldwide, solutions to reduce or eliminate their interconnected impacts on diet-related NCDs require integrated approaches at multiple scales (global, national, and local). According to previous research, solutions for the prevention of livestock diet-related NCDs are largely divided into political commitments (e.g., poverty alleviations) [11, 62] and individual lifestyle changes (e.g., dietary change toward Mediterranean and vegetarian diets) [1, 4]. International organizations such as the FAO, WHO, and UNEP can combine multiple separate solutions for NCDs’ reduction in integrated initiatives or agreements. The UN’s Sustainable Development Goals are a good example of such integrated solutions for NCDs’ reduction [9].

Additionally, national-level solutions should consider socioeconomic inequalities to implement effective human health policies for NCDs’ reduction [2, 11]. With the increase in livestock imports, decreases in prices of unhealthy animal products (e.g., fatty and processed meat) can exacerbate diet-related NCD risks, particularly in poor populations [11, 18]. Furthermore, in developing countries, malnutrition and poverty exacerbated by these inequalities hobbled those countries’ attempts to construct adaptable health policies for managing NCD risks. Local communities and individuals play an important role in achieving global sustainability, because they can alter their diets to consume fewer livestock products [1, 4, 63, 64]. National policies help stimulate national-level changes with effective financial mechanisms such as subsidies and taxes for livestock commodities [59, 65, 66]. Strengthening policy coherence at different scales of policies has a positive influence on achieving sustainable development regarding NCDs (e.g., SDG #3 – good health and well-being).

Before drawing conclusions, we should be cautious regarding the limitations of our study. First, in our path analyses, we could not fully determine causal directions of the interrelationships among livestock production, trade, consumption, and NCD risks. Nevertheless, we feel our path analysis model captures the dominant interrelationships between livestock import activities and diet-related NCD risks and lays the groundwork for future research. Further work is required to examine causality in changes in diet-related NCD risks due to livestock trade (e.g., using a comparative risk assessment). Second, we identified a few developed countries that did not follow the trends identified in our path analyses, which may weaken our results. For example, Saudi Arabia and South Korea (developed and non-western countries) both had low livestock consumption (249.6 and 278.3 kcal/capita/day, respectively) and livestock diet-related NCD DALY rates (1.7 and 42.1 years per 100,000 people). Third, although our results may indicate that healthcare infrastructure and NCD policies contributed to diet-related NCD risks and deaths, we could not statistically detect these contributions because of the lack of global datasets. For example, health care systems with varied affordability may also lead to different NCD mortality in the UK and USA [67]. Future research will need to include the scores of healthcare infrastructure and NCD policies at the country level. Furthermore, future research should also evaluate the role of other agents (e.g., traders, policymakers, and processors) that can affect livestock trade, consumption, and NCD risks, in addition to the producers and consumers included in this study.

## Conclusions

The interconnections among livestock production, trade, and consumption are telecoupling processes that can threaten human health around the world. By using the telecoupling framework, this research provides scientific evidence of these interconnections and their contributions to livestock diet-related NCDs simultaneously. We determined that livestock imports are positively associated with rising diet-related NCDs via the increases in livestock consumption, particularly in developing countries. In our tightly telecoupled world, global changes in livestock production, trade, and consumption are associated with the abrupt development of diet-related health problems, especially in developing countries. These nations, which were until recently wrestling with poverty and malnutrition, are now being blindsided by rapid increases in chronic diet-related NCD risks. Understanding these interconnections among livestock production, trade, and consumption, and tapping into lessons learned from neighboring and distant trade partners, can help mitigate diet-related NCD risks. Thus, new governance structures for the incorporation of separate international efforts, improved national policies, and supporting individual efforts are required to reduce diet-related NCDs driven by the livestock trade.

## Additional files

Additional file 1: List of developed and developing countries (DOCX 14 kb)

Additional file 2: Detailed list of crop and livestock products (DOCX 14 kb)

Additional file 3: Path analysis of the relationships between meat consumption and diet-related NCD risks through meat production and trade from 1992 to 2011 (DOCX 18 kb)

Additional file 4: Crop supply by use: (A) Brazil, (B) China, (C) the UK, and (D) the USA (DOCX 75 kb)

## Abbreviations

CFI: Comparative fit index
DALYs: Disability-adjusted life years
GHDx: Global health data exchange
ML: Maximum likelihood
NCDs: Non-communicable diseases
SDGs: Sustainable development goals
SRMR: Standardized root mean square residual
UN: United Nations
WHO: World Health Organization
