# Supplementary material for: Telecoupled impacts of livestock trade on non-communicable diseases
Source: Global Health. 2019 Jul 1;15:43. doi: 10.1186/s12992-019-0481-y (PMC6604153; doi:10.1186/s12992-019-0481-y)
Supplement: Supplementary file 1 — List of developed and developing countries (DOCX 14 kb) [file 12992_2019_481_MOESM1_ESM.docx]

Additional file 1 List of developed and developing countries

| **Developed countries** (n=46) |
| --- |
| Antigua and Barbuda, Australia, Austria, Bahamas, Barbados, Belgium, Brunei Darussalam, Canada, Chile, Croatia, Cyprus, Czech Republic, Denmark, Estonia, Finland, France, Germany, Greece, Iceland, Ireland, Israel, Italy, Japan, Latvia, Lithuania, Luxembourg, Netherlands, New Zealand, Norway, Oman, Poland, Portugal, Republic of Korea, Russian Federation, Saint Kitts and Nevis, Saudi Arabia, Slovakia, Slovenia, Spain, Sweden, Switzerland, Trinidad and Tobago, United Kingdom of Great Britain and Northern Ireland, United Arab Emirates, United States of America, Uruguay |
| **Developing countries** (n=110) |
| Afghanistan, Albania, Algeria, Angola, Armenia, Azerbaijan, Bangladesh, Belarus, Belize, Benin, Bolivia (Plurinational State of), Bosnia and Herzegovina, Botswana, Brazil, Bulgaria, Burkina Faso, Cambodia, Cameroon, Cabo Verde, Central African Republic, Chad, China, Colombia, Congo, Costa Rica, Cuba, Cote d'Ivoire, Djibouti, Dominica, Dominican Republic, Ecuador, El Salvador, Ethiopia, Fiji, Gabon, Gambia, Georgia, Ghana, Grenada, Guatemala, Guinea, Guinea-Bissau, Guyana, Haiti, Honduras, Hungary, India, Indonesia, Iran (Islamic Republic of), Iraq, Jamaica, Kazakhstan, Kenya, Kyrgyzstan, Lao People's Democratic Republic, Lebanon, Lesotho, Liberia, Madagascar, Malawi, Malaysia, Mali, Mauritania, Mauritius, Mexico, Mongolia, Montenegro, Morocco, Mozambique, Namibia, Nepal, Nicaragua, Niger, Nigeria, Pakistan, Panama, Paraguay, Peru, Philippines, Republic of Moldova, Romania, Rwanda, Saint Lucia, Saint Vincent and the Grenadines, Samoa, Senegal, Serbia, Sierra Leone, Solomon Islands, South Africa, Sri Lanka, Suriname, Swaziland, Tajikistan, Thailand, The former Yugoslav Republic of Macedonia, Togo, Tunisia, Turkey, Turkmenistan, Uganda, Ukraine, United Republic of Tanzania, Uzbekistan, Vanuatu, Venezuela (Bolivarian Republic of), Viet Nam, Yemen, Zambia, Zimbabwe |
