# Supplementary material for: Telecoupled impacts of livestock trade on non-communicable diseases
Source: Global Health. 2019 Jul 1;15:43. doi: 10.1186/s12992-019-0481-y (PMC6604153; doi:10.1186/s12992-019-0481-y)
Supplement: Supplementary file 2 — Detailed list of crop and livestock products (DOCX 14 kb) [file 12992_2019_481_MOESM2_ESM.docx]

Additional file 2 Detailed list of crop and livestock products

| **Crop Products** |
| --- |
| Apples, Bananas, Barley, Beans, Brans, Cassava, Cereals - Other, Cocoa Beans, Coconuts - Incl, Copra, Copra Cake, Cottonseed, Cottonseed Cake, Dates, Fruits - Other, Grapes, Groundnut Cake, Groundnuts (Shelled Eq), Maize, Millet, Molasses, Oats, Oilcrops Oil, Other, Oilcrops, Other, Oilseed Cakes, Other, Olive Oil, Onions, Oranges - Mandarines, Palmkernel Cake, Palmkernels, Peas, Plantains, Potatoes, Pulses - Other, Rape and Mustard Cake, Rape and Mustard Oil, Rape and Mustardseed, Rice (Milled Equivalent), Roots - Other, Rye, Sesameseed, Sesameseed Cake, Sorghum, Soyabean Cake, Soyabean Oil, Soyabeans, Sugar (Raw Equivalent), Sugar Beet, Sugar Cane, Sugar - Non-Centrifugal, Sunflowerseed, Sunflowerseed Cake, Sweet Potatoes, Sweeteners - Other, Tomatoes, Vegetables - Other, Wheat, Yams |
| **Livestock Products** |
| Bacon and ham, Camels fat, Cattle fat, Liver prepared (foie gras) fat, nes prepared fat, Pigs fat, Beef and veal sausages meat, Beef preparations meat, Cattle boneless (beef & veal)meat, Chicken meat, Chicken canned meat, Dried nes meat, Duck meat, Game meat, Goat meat, Goose and guinea fowl meat, Horse meat, nes meat, Pig meat, Pig sausages meat, Pig preparations meat, Pork meat, Rabbit meat, Turkey meat, Cattle edible offals, Goats edible offals, Liver chicken offals, Liver duck offals, Liver geese offals, Other camelids offals, Pigs edible offals, Sheep edible offals, Fats of animal nes oils |
