# Supplementary material for: Telecoupled impacts of livestock trade on non-communicable diseases
Source: Global Health. 2019 Jul 1;15:43. doi: 10.1186/s12992-019-0481-y (PMC6604153; doi:10.1186/s12992-019-0481-y)
Supplement: Supplementary file 3 — Path analysis of the relationships between meat consumption and diet-related NCD risks through meat production and trade from 1992 to 2011 (DOCX 18 kb) [file 12992_2019_481_MOESM3_ESM.docx]

Additional file 3 Path analysis of the relationships between meat consumption and diet-related NCD risks through meat production and trade from 1992 to 2011

| **Path analysis** | **Unstandardized coefficient (S.E.)** |
| --- | --- |
| **Dependent variable: Meat production (kcal)** | |
| Protein supply of animal origin (g/capita/day) | 1.482** (0.185) |
| Pastures and meadows areas (km^2^) | **–**0.068* (0.031) |
| GDP-PPP per capita (2011 $ const.) | **–**0.006 (0.103) |
| Population (persons) | 1.190** (0.048) |
| **Dependent variable: Meat export (kcal)** | |
| Protein supply of animal origin (g/capita/day) | 4.368** (1.210) |
| Pastures and meadows (km^2^) | **–**0.060 (0.203) |
| GDP-PPP per capita (2011 $ const.) | 1.425* (0.677) |
| Population (persons) | 1.475** (0.313) |
| **Dependent variable: Meat import (kcal)** | |
| Protein supply of animal origin (g/capita/day) | 0.918* (0.395) |
| Pastures and meadows (km^2^) | **–**0.004 (0.066) |
| GDP-PPP per capita (2011 $ const.) | 0.968** (0.221) |
| Population (persons) | 0.472** (0.102) |
| **Dependent variable: Meat consumption (kcal/capita/day)** | |
| Meat Production (kcal) | 0.366** (0.036) |
| Meat Export (kcal) | **–**0.003 (0.006) |
| Meat Import (kcal) | 0.038* (0.018) |
| Pastures and meadows (km^2^) | 0.062** (0.015) |
| GDP-PPP per capita (2011 $ const.) | 0.223** (0.044) |
| Population (persons) | **–**0.547** (0.043) |
| **Dependent variable: Disability-adjusted life years (years)** | |
| Meat consumption (kcal/capita/day) | 1.374** (0.175) |
| Pastures and meadows (km^2^) | 0.016 (0.046) |
| GDP-PPP per capita (2011 $ const.) | 0.664** (0.118) |
| Population (persons) | 1.110** (0.071) |
| **Dependent variable: Number of deaths from diets high in meat (persons)** | |
| Meat consumption (kcal/capita/day) | 1.413** (0.183) |
| Pastures and meadows (km^2^) | **–**0.018 (0.048) |
| GDP-PPP per capita (2011 $ const.) | 0.563** (0.124) |
| Population (persons) | 1.002* (0.075) |
| **Dependent variable: Age-standardized death rate from diets high in meat (per 100,000 people)** | |
| Meat consumption (kcal/capita/day) | 0.478** (0.100) |
| Pastures and meadows (km^2^) | 0.015 (0.026) |
| GDP-PPP per capita (2011 $ const.) | 0.262** (0.068) |
| Population (persons) | 0.083* (0.041) |
| $\chi^{2}$ | 154.253 |
| df | 14 |
| CFI | 0.937 |
| SRMR | 0.028 |

Values in parentheses are standard errors.

# All variables are log transformation variables, except the index of NCD risk factors.

* P<0.05, ** P<0.001
