# Supplementary material for: Telecoupled impacts of livestock trade on non-communicable diseases
Source: Global Health. 2019 Jul 1;15:43. doi: 10.1186/s12992-019-0481-y (PMC6604153; doi:10.1186/s12992-019-0481-y)
Supplement: Supplementary file 4 — Crop supply by use: (A) Brazil, (B) China, (C) the UK, and (D) the USA (DOCX 75 kb) [file 12992_2019_481_MOESM4_ESM.docx]

Additional file 4 **Crop supply by use.** **(A)** Crops in **Brazil** were increasingly used for livestock feed, processing, and other uses. **(B)** In **China**, food supply and livestock feed contributed to increase total crop supply. China used more crop calories for food supply than livestock feed. **(C)** The **UK** stabilized total crop supply and used higher crop calories for livestock feed. **(D)** The **USA** increased crop supply for processing and other uses after 1970. Data Source: [42].
